# Sexually transmitted infections in Saudi Arabia

**DOI:** 10.1186/1471-2334-6-3

**Published:** 2006-01-10

**Authors:** Tariq A Madani

**Affiliations:** 1 Department of Medicine, King Abdulaziz University, Jeddah, and Ministry of Health, Riyadh, Saudi Arabia

## Abstract

**Background:**

Data on sexually transmitted infections (STIs) in Saudi Arabia (SA) and other Islamic countries are limited. This study describes the results of a five-year surveillance for STIs in SA.

**Methods:**

This is a case series descriptive study of all confirmed STIs diagnosed in SA from January, 1995 through December, 1999.

**Results:**

A total of 39049 STIs were reported to the Ministry of Health. Reported STIs included nongonococcal urethritis (14557 infections, 37.3%), trichomoniasis (10967 infections, 28.1%), gonococcal urethritis (5547 infections, 14.2%), syphilis (3385 infections, 8.7%), human immunodeficiency virus (2917 infections, 7.5%), genital warts (1382, 3.5%), genital herpes (216 infections, 0.6%), and chancroid (78 infections, 0.2%). The average annual incidence of STIs per 100,000 population for Saudis and non-Saudis, respectively, was as follows: 14.8 and 7.5 for nongonococcal urethritis, 9.4 and 10.4 for trichomoniasis, 5.2 and 4.2 for gonorrhea, 1.7 and 6.4 for syphilis, 0.6 and 8.0 for HIV, 1.4 and 0.7 for genital warts, 0.1 and 0.4 for genital herpes, and 0.1 and 0.1 for chancroid. The incidence of STIs was somewhat steady over the surveillance period except for nongonococcal urethritis which gradually increased.

**Conclusion:**

Nongonococcal urethritis, trichomoniasis, and gonococcal urethritis were the most commonly reported STIs in SA. Even though the incidence of STIs in SA is limited, appropriate preventive strategies that conform to the Islamic rules and values are essential and should be of highest priority for policymakers because of the potential of such infections to spread particularly among the youth.

## Background

Sexually transmitted infections (STIs) are one of the most under-recognized health problems worldwide. While extremely common, STIs are difficult to track. Many people with these infections do not have symptoms and remain undiagnosed. Further, diseases that are diagnosed are frequently not reported and counted. Most of the published data on the prevalence and incidence of STIs come from developed countries.

Despite the tracking difficulties, the estimated global annual incidence of curable STIs (excluding HIV and viral hepatitis) is 333 million cases; gonococcal infections, 62 million cases, chlamydial infections, 89 million cases, syphilis, 12 million cases, and trichomoniasis, 170 million cases [1]. In the United States, estimates in 1999 indicated that more than 65 million people were living with incurable STIs and that 15 million people become infected with one or more STI each year, roughly half of whom contract lifelong infections [2]. Approximately one-fourth of these new infections were in teenagers. While some STIs, such as syphilis, were brought to all time lows, others, like genital herpes, gonorrhea, and chlamydia, continued to resurge and spread through the population in the United States [3].

Information about STIs in Islamic countries, where non-marital sex and homosexuality are prohibited by religion, is notably limited. An assumed low prevalence of STIs and religious and cultural intolerability of non-marital sex and homosexuality in Islamic countries are expected reasons for the limited data. Detailed information on human immunodeficiency virus (HIV) in Saudi Arabia (SA) was recently published for the first time from such a country [4]. However, data on other STIs from this Islamic country have not been published. This study describes the results of surveillance activities for STIs that have been underway in SA from January, 1995 through December, 1999, and the preventive strategies adopted by the country.

## Methods

### Saudi Arabia

SA occupies most of the Arabian Peninsula with an area of about 2,240,000 sq km. The latest census conducted in SA in 2004 indicated that the total population is 22,673,538. The Saudi citizen population is 16,529,302 (72.9% of the total population); 50.1% (8,285,662 people) of them are males and 49.9% (8,243,640 people) of them are females. The non-citizen population is 6,144,236 (27.1% of the total population); 69.5% (4271598 people) of them are males and 30.5% (1,872,638 people) of them are females. Approximately, 42.3% of the population is below 15 years of age, 54.8%, between 15 and 64 years, and 2.9%, above 64 years of age. All Saudi citizens and most of the non-Saudi nationals are Muslims and the country is governed according to the Islamic law.

### Data collection

HIV has been notifiable in SA since 1984. Reporting other STIs has been mandated by the Ministry of Health (MOH) in SA since 1995. The MOH officials rely on health-care providers, laboratories, and other public health personnel to report the occurrence of STIs to the Department of Preventive Medicine in the Central MOH office in Riyadh where all surveillance data are compiled. During this study period, from January, 1995 through December, 1999, annual reports were produced but they were only utilized internally by the concerned officials in the MOH and the Ministry of Interior and they were not made available for the public. Since year 2000, the concerned officials decided to make data on all STIs available for the public as an essential part of health educational campaigns to increase the public's awareness of the prevalent STIs in SA for preventive purposes.

### Case definitions

The case definition of STIs used during the surveillance period was adopted from the case definitions published by the Centers for Disease Control and Prevention (CDC) [5]. Chancroid was defined as any painful genital ulceration and inflammatory inguinal lymphadenopathy with isolation of *Haemophilus ducreyi *from the ulcer discharge. Gonococcal urethritis was defined as any abnormal urethral discharge with gram negative diplococci seen on Gram stain of the urethral discharge or isolation of *Neisseria gonorrhoeae *from it. Nongonococcal urethritis was defined as any abnormal urethral discharge that was not the result of infection with *Neisseria gonorrhoeae*. Genital herpes was defined as any painful genital or anal lesions with either a history of one or more previous episodes of similar genital lesions or isolation of *Herpes simplex *virus HSV from the cervix, urethra, or anogenital lesion, or demonstration of virus by antigen detection technique in clinical specimens from cervix, urethra, or anogenital lesion, or demonstration of multinucleated giant cells on a Tzanck smear of scrapings from an anogenital lesion. Genital warts were defined as the presence of painless exophytic (raised) growths on the internal or external genitalia, perineum, or perianal region with histopathologic changes characteristic of human papillomavirus infection in specimens obtained by biopsy or exfoliative cytology or demonstration of virus by antigen or nucleic acid detection in a lesion biopsy. HIV infection was defined as any patient with positive HIV antibodies detectable by ELISA and confirmed by Western blot test. Syphilis was defined as an illness or state of health compatible with primary, secondary, latent, or tertiary syphilis with either demonstration of *Treponema pallidum *in clinical specimens (by darkfield microscopy or direct fluorescent antibody) or a reactive nontreponemal serologic test (Venereal Disease Research Laboratory [VDRL] or rapid plasma reagin [RPR]) and a reactive specific treponemal serologic test (*Treponema pallidum *hemagglutination test [TPHA] or fluorescent treponemal antibody absorbed [FTA-ABS]). Trichomoniasis was defined as the demonstration of *Trichomonas vaginalis *on microscopical examination of urethral or vaginal discharge. The MOH mandated that genital herpes and genital warts should be reported only once per patient regardless of the number of recurrent episodes.

## Results

**Figure 1:**
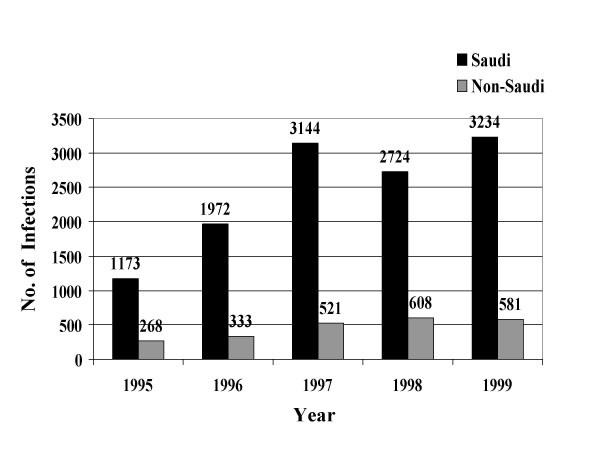
Annually reported number of non-gonococcal urethritis infections in Saudi Arabia from 1995 to 1999.

From January, 1995 through December, 1999, a total of 39049 STIs were reported to the MOH. Reported STIs included nongonococcal urethritis, trichomoniasis, gonococcal urethritis, syphilis, HIV, genital warts, genital herpes, and chancroid. Table 1 shows the total number and average annual incidence of STIs per 100,000 population by citizenship. Figures 1 through 8 show the annual incidence of each STI during the surveillance period.

**Figure 2:**
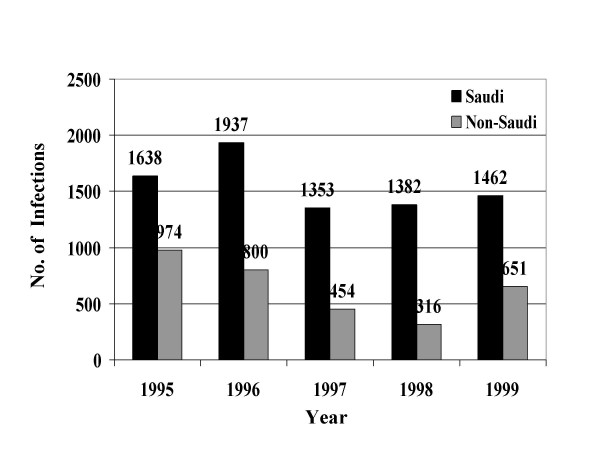
Annually reported number of trichomoniasis infections in Saudi Arabia from 1995 to 1999.

**Figure 3:**
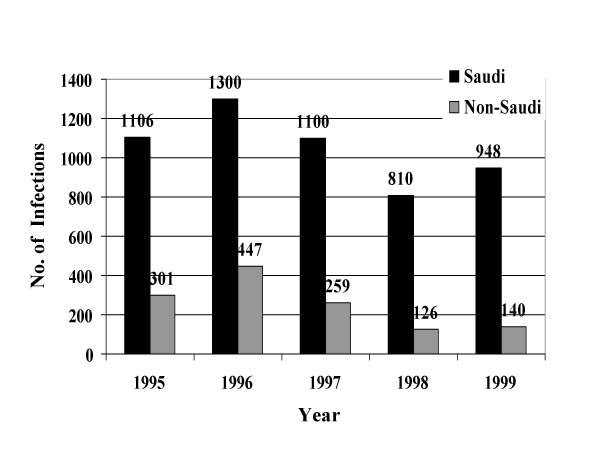
Annually reported number of gonorrhea infections in Saudi Arabia from 1995 to 1999

**Figure 4:**
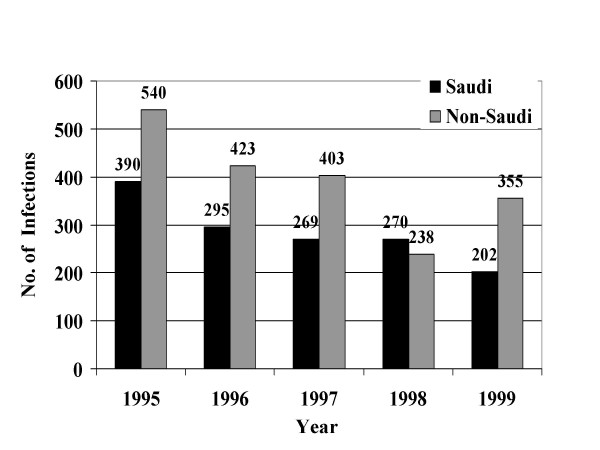
Annually reported number of syphilis infections in Saudi Arabia from 1995 to 1999

**Figure 5:**
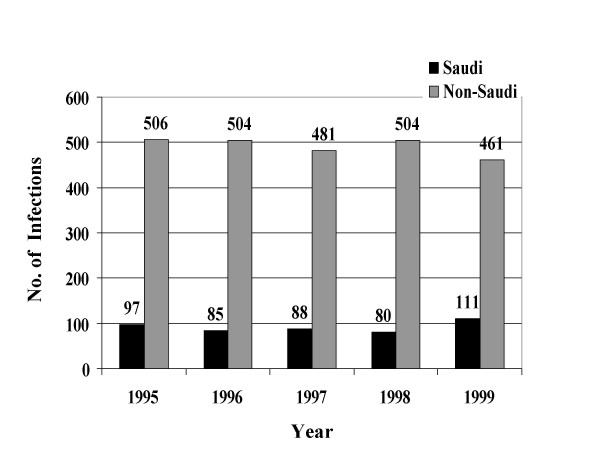
Annually reported number of HIV infections in Saudi Arabia from 1995 to 1999.

**Figure 6:**
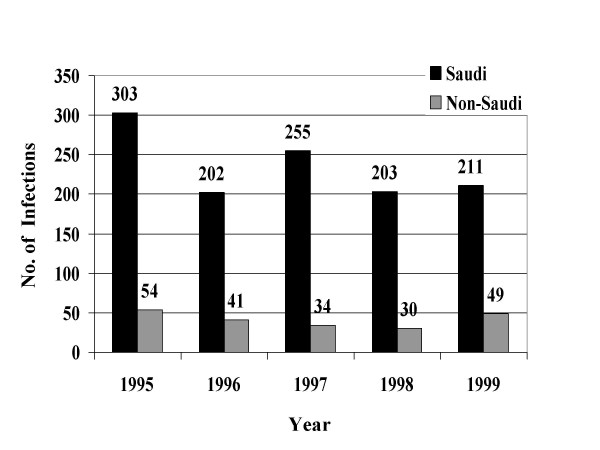
Annually reported number of genital warts infections in Saudi Arabia from 1995 to 1999.

**Figure 7:**
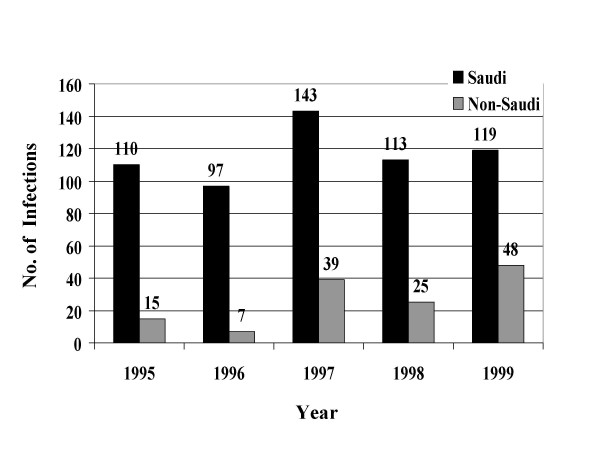
Annually reported number of genital herpes infections in Saudi Arabia from 1995 to 1999.

**Figure 8:**
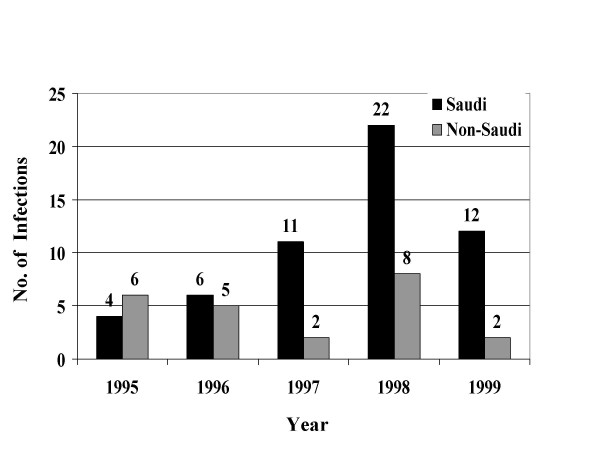
Annually reported number of chancroid infections in Saudi Arabia from 1995 to 1999.

## Discussion

Data on STIs in SA and other Islamic countries are very limited. Detailed information on the epidemiology of HIV infection in SA has recently been published [4]. However, data on other STIs in SA have not been published. The current study described the incidence of STIs in SA over a five-year period of surveillance. The incidence of STIs in SA was found to be low when compared to other countries such as the United States. For example, in 1999, the average incidence of gonorrhea per 100,000 population in the United States was 131.4, whereas, in SA, it was 4.9 [3]. It is estimated that 5.5 million cases of genital warts, 5 million cases of trichomoniasis, 3 million cases of nongonococcal urethritis, 1 million cases of herpes, 650,000 cases of gonorrhea, and 70,000 cases of syphilis are reported every year in the United States [3]. It is possible that the data in SA, as the case in the United States, underrepresented the actual magnitude of STIs in this country because of the possibility of underreporting. In the United States, for example, the cases of gonorrhea reported to the CDC are believed to represent about half of the annual infections [3]. However, while an underestimate of actual cases, these data provide a good indication of trends in STIs. Of note, however, that the HIV data likely closely represented the actual magnitude of HIV in SA during the surveillance period because of the known good adherence of health-care providers and laboratories to notification of HIV infection in particular [4].

The incidence of STIs varied widely between Saudis and non-Saudis. Nongonococcal and gonococcal urethritis and genital warts were more frequently reported among Saudis, whereas HIV, syphilis, and genital herpes were more frequently reported among non-Saudis. One possible explanation of the higher incidence of HIV and syphilis among non-Saudi population is the fact that all non-Saudi workers are routinely screened for these infections pre-employment and every two years, thereafter, to have their legal residence permits renewed. Generally, the incidence of STIs had been somewhat steady over the surveillance period except for nongonococcal urethritis which had gradually increased.

The impact of adhering to Islamic values on the prevalence of STIs was demonstrated by several studies. According to the United Nations and the World Health Organization data on HIV prevalence in different countries, the prevalence of HIV infection in Islamic countries is strikingly low compared to other countries [6,7]. A recent study showed that among 38 sub-Saharan African countries, the percentage of Muslims within countries negatively predicted HIV prevalence [8]. A survey of published journal articles containing data on HIV prevalence and religious affiliation showed that six of seven such studies indicated a negative relationship between HIV prevalence and being Muslim [8]. It should be noted, however, that the preventive strategies in some Islamic countries do not necessarily abide by the Islamic doctrine and that knowledge, attitude, and practice of Muslims in various Islamic societies do not necessarily conform to Islamic norms.

Some of the STIs preventive strategies that are advocated and used in non-Islamic countries are not acceptable in Islamic countries. For instance, the concept of "Safe Sex" to prevent STI in non-Islamic countries basically promotes the use of condoms for non-marital sexual relations, considered in Islamic countries a way of promoting non-marital sex which is absolutely prohibited in Islam. The concept of safe sex in Islam implies monogamous sexual relationship through legal marriage. Similarly, needle exchange programs advocated in non-Islamic countries as a means to prevent HIV and other blood-borne infections, is viewed by Islam as a way of encouraging people engaged in intravenous drug use to continue this prohibited practice. Such programs, therefore, can not be accepted by Muslim communities.

Strategies to prevent STIs in Islamic countries have to abide by the Islamic rules and values and should include strengthening of Islamic and health education, encouraging people to follow and implement the Islamic rules and values that prohibit adultery and homosexuality, and to practice safe sex only through legal marriage. Helping the youth to get married and reducing the cost of marriage are also strongly recommended in Islam and should be the responsibility of both governmental and non-governmental charitable organizations and the population at large. There are several charitable programs in SA successfully helping thousands of young men and women to get married with the cost entirely covered by donations.

Other aspects in Islam to prevent non-marital sex include allowing men to be married to up to four women and permitting adolescents to get married with no age limit for marriage. Additionally, Islam obliges women to cover themselves with veils (Hijabs) and to be segregated from men in educational institutes and other gathering places to prevent provocation of men. Islam also fights poverty, a driving force for commercial sex and prostitution, through a well established system of obligatory charity, known as "Zakat", and voluntary charity, known as "Sadaqa", taken from the rich people and given to the poor and needy. Additionally, Islam obliges the rulers to eliminate all means and factors that are conducive to indulging in non-marital sex and intravenous drug use such as sex trade and prostitution and to implement the Islamic penalties on those involved in such illegal acts. The penalties for those who commit adultery (non marital sex between a woman and a man) range from just whipping (for those who are not married) to execution (for those who are married). However, these penalties can only be implemented if the act of illegal sexual intercourse was witnessed by four people, which is practically almost impossible. The penalty for adults involved in homosexuality is execution of both partners regardless of whether they are married to women. Again, the actual act of anal intercourse has to be witnessed by four people for the adult partners to be sentenced to death. Such penalties, albeit rarely implemented because of the conditions that need to be fulfilled, are scary enough to make most people with weak faith to stay away from adultery and homosexuality. The penalties for drug abuse involve whipping and incarceration and do not reach to execution. In SA, detoxification and treatment are also always offered to drug addicts in special detoxification centers. The penalties for those involved in drug smuggling are vast but in SA, it may reach up to execution.

Other strategies to prevent STIs in SA include health education, early diagnosis and treatment, contact tracing, and routine screening of blood and organ donors, pregnant women, newborns of infected mothers, prisoners, intravenous drug users, patients with other sexually transmitted infections, and expatriates pre-employment for HIV, syphilis, and viral hepatitis B and C. Partners of patients with STIs are informed and counseled on the appropriate preventive measures and the required tests and, when necessary, treatment.

Partners of patients with nongonococcal urethritis, trichomoniasis, or gonococcal urethritis are empirically treated for these infections. HlV-negative partners of HIV-positive patients are instructed to use condoms for sexual intercourse. Non-immune hepatitis B-negative partners of hepatitis B-positive subjects are routinely vaccinated with the hepatitis B vaccine. Thus, religious and medical means are used in concert to prevent STIs in SA.

**Table 1:** Total number and average annual incidence of sexually transmitted infections per 100,000 population in Saudi Arabia from 1995 to 1999.

Infection	Number of infections among Saudi patients (%)	Average annual incidence of infections per 100,000 Saudi population	Number of infections among non-Saudi patients (%)	Average annual incidence of infections per 100,000 non-Saudi population	Total number of infections (%)	Average annual incidence of infections per 100,000 total population
Nongonococcal urethritis	12246 (84.1)	14.8	2311 (15.9)	7.5	14557 (37.3)	12.8
Trichomoniasis	7772 (70.9)	9.4	3195 (29.1)	10.4	10967 (28.1)	9.7
Gonococcal urethritis	4274 (77.1)	5.2	1273 (22.9)	4.2	5547 (14.2)	4.9
Syphilis	1426 (42.1)	1.7	1959 (57.9)	6.4	3385 (8.7)	3.0
Human immunodeficiency virus	461 (15.8)	0.6	2456 (84.2)	8.0	2917 (7.5)	2.6
Genital warts	1174 (84.9)	1.4	208 (15.1)	0.7	1382 (3.5)	1.2
Genital herpes	82 (38.0)	0.1	134 (62.0)	0.4	216 (0.6)	0.2
Chancroid	55 (70.5)	0.1	23 (29.5)	0.1	78 (0.2)	0.1
Total	27490 (70.4)	32.3	11559 (29.6)	37.6	39049	34.4

## Conclusion

Nongonococcal urethritis, trichomoniasis, and gonococcal urethritis were the most commonly reported STIs in SA. Even though the incidence of STIs in SA is limited, appropriate preventive strategies that conform to the Islamic rules and values are essential and should be of highest priority for policymakers because of the potential of such infections to spread particularly among the youth.

## Competing interests

The author(s) declares that he has no competing interests.

## Pre-publication history

The pre-publication history for this paper can be accessed here:



## References

[B1] World Health Organization (WHO) (1995). An Overview of Selected Curable Sexually Transmitted Diseases.

[B2] Cates W (1999). Estimates of the Incidence and Prevalence of Sexually Transmitted Diseases in the United States. American Social Health Association Panel. Sex Transm Dis.

[B3] (2000). Tracking the hidden epidemics. Trends in STDs in the United States CDC.

[B4] Madani TA, Al-Mazrou YY, Al-Jeffri MH, Al-Huzaim NS (2004). Epidemiology of the human immunodeficiency virus in Saudi Arabia; 18-year surveillance results and prevention from an Islamic perspective. BMC Infectious Diseases.

[B5] CDC (1990). Case definitions for public health surveillance, 1990. MMWR.

[B6] United Nations Programme on HIV/AIDS (UNAIDS) (2002). Report on the Global HIV/AIDS Epidemic.

[B7] Lenton C (1997). Will Egypt escape the AIDS epidemic?. Lancet.

[B8] Gray PB (2004). HIV and Islam: is HIV prevalence lower among Muslims?. Soc Sci Med.

